# COVID-19 vaccination is associated with enhanced efficacy of anti-PD-(L)1 immunotherapy in advanced NSCLC patients: a real-world study

**DOI:** 10.1186/s13027-023-00526-7

**Published:** 2023-09-07

**Authors:** Yunfei Qian, Zhuxian Zhu, Yin-Yuan Mo, Ziqiang Zhang

**Affiliations:** 1 Department of Respiratory and Critical Care Medicine, Tongji University School of Medicine, Shanghai, China; 2 Department of Nephrology, Tongji Hospital, Tongji University School of Medicine, Shanghai, China; 3 Institute of Clinical Medicine, Zhejiang Provincial People’s Hospital of Hangzhou Medical College, Hangzhou, China; 4 Department of Respiratory and Critical Care Medicine, Shanghai Pudong Hospital, Fudan University Pudong Medical Center, Pudong Hospital of Fudan University, Shanghai, China

**Keywords:** COVID-19 vaccination, Immune checkpoint inhibitor (ICI), Non-small cell lung cancer (NSCLC), Progression-free survival (PFS), Overall survival (OS)

## Abstract

**Background:**

Coronavirus disease 2019 (COVID-19) vaccine has played a major role in ending the pandemic. However, little is known about the influence of COVID-19 vaccine on the efficacy of immunotherapy in patients with non-small cell lung cancer (NSCLC).

**Objectives:**

The goal of this study is to explore whether COVID-19 vaccine impacts the efficacy of immune checkpoint inhibitors (ICIs) in NSCLC patients.

**Methods:**

We retrospectively analyzed the survival data of ICI-treated 104 patients with stage III–IV NSCLC, who either received COVID-19 vaccination (n = 25) or no vaccination (n = 79). The potential risk factors, in particular roles of COVID-19 vaccination in the efficacy of ICIs in these patients, were evaluated.

**Results:**

Our results showed significantly improved ORR (28.0% vs. 11.39%, *p* = 0.05) and DCR (88.0% vs. 54.43%, *p* = 0.005) in the COVID-19 vaccinated group compared with the non-vaccinated group. Regarding the long-term survival benefits, COVID-19 vaccine showed profound influence both on the PFS (HR = 0.16, *p* = 0.021) and OS (HR = 0.168, *p* = 0.019) in patients with NSCLC under ICIs treatment. The PFS (*p* < 0.001) or OS (*p* < 0.001) was significantly improved in the COVID-19 vaccinated group, compared with the non-vaccinated group. Moreover, CD4 T cell (*p* = 0.047) level was higher in the COVID-19 vaccinated group than in the non-vaccinated group.

**Conclusions:**

COVID-19 vaccination enhances anti-PD-1 immunotherapy efficacy in patients with stage III–IV NSCLC, suggesting that COVID-19 vaccination may provide additional benefit to NSCLC patients.

## Introduction

Lung cancer is the leading cause of cancer death worldwide [1]. A study showed that, among approximately 60,000 patients receiving antitumor treatment, lung cancer patients had the highest incidence of COVID-19 [2]. Studies [3, 4] also have shown that cancer patients are more susceptible to COVID-19. Moreover, NSCLC patients may suffer from fatal clinical outcomes of COVID-19. The mortality of lung cancer patients was higher than the control or other malignant tumor patients [5]. In addition, cancer patients with COVID-19 have a higher risk of tracheal intubation, intensive care unit admission and death [6].

Immune checkpoint inhibitors (ICIs) have demonstrated promising therapeutic effects and brought long-lasting objective remission in some NSCLC patients [7–9]. Interestingly, anticancer therapies, such as chemotherapy, were associated with higher 30-day all-cause mortality due to COVID-19, while immunotherapy (such as immune checkpoint inhibitors) was not associated with higher COVID-19 disease severity [10].

Studies have further shown that COVID-19 vaccination is safe in cancer patients including lung cancer [11–14]. Thus, active immunization of COVID-19 vaccines has been suggested for cancer patients to prevent COVID-19 [15, 16]. A meta-analysis showed that COVID-19 vaccine is effective and safe in cancer patients receiving ICI [17]. More importantly, COVID-19 vaccination was generally well tolerated, and did not seem to increase the incidence of immune-related adverse events (irAE) in cancer patients who received ICIs [18]. For example, among 2,134 nasopharyngeal cancer patients who received anti-PD-1 treatment, COVID-19 vaccinated patients showed a higher objective response rate and disease control rate [19]. Similarly, COVID-19 vaccinated cancer patients following camrelizumab treatment were more likely to be in better conditions and experienced stable disease [20].

However, it is not clear whether COVID-19 vaccination has any benefit for NSCLC patients who are under ICIs treatment. Herein, we tried to address this question in advanced lung cancer patients at stage III–IV NSCLC who received anti-PD-(L)1 immunotherapy.

## Patients and methods

### Patient information

**Fig. 1:**
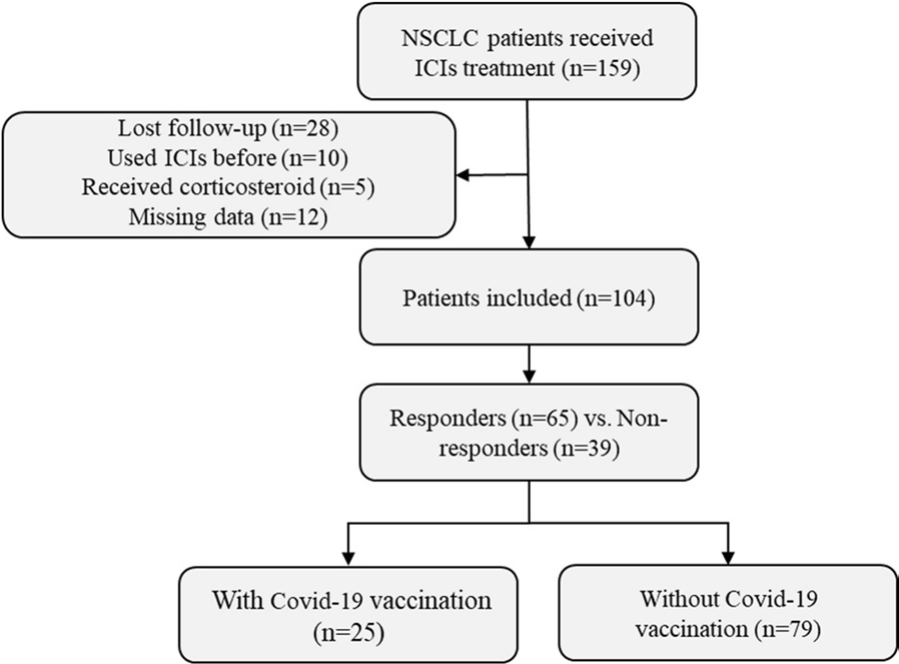
Flow diagram of this study

We screened a total of 159 lung cancer patients in this retrospective study and selected a cohort of 104 patients with initially diagnosed of stage III–IV NSCLC (Fig. 1). All the patients were from Tongji Hospital of Tongji University in Shanghai, China from August 2018 to August 2022, who received ICIs. All participants were evaluated by computed tomography or magnetic resonance imaging using Response Evaluation Criteria in Solid Tumors version 1.1 (RECIST 1.1). ICIs used in this study included pembrolizumab, tislelizumab, toripalimab, camrelizumab, or sintilimab. Patients` inclusion criteria are as follows: (i) All patients were diagnosed by cytological and/or histological examination according to the WHO classification; (ii) the stage of lung cancer was stage III–IV; (iii) lung cancer patients received ICIs for the first line or subsequent lines treatment; (iv) patients received COVID-19 vaccine before ICIs treatment in the COVID-19 vaccination group. Exclusion criteria: (i) patients suffered from other types of malignant tumors; (ii) patients had autoimmune disorders; (iii) patients received systemic corticosteroid treatment.

### Clinical data collection

Patient demographics and clinical characteristics in this study included age, gender, ECOG value, COVID-19 vaccination, tumor stage, pathological type of tumor, and smoking status. Laboratory testing included white blood cell count, neutrophil count, lymphocyte count, C-reactive protein, platelet count, D-dimer and T cells. All procedures performed in this study followed the Declaration of Helsinki (as revised in 2013).

### Clinical outcomes and ICIs response assessment

The response of ICIs treatment was evaluated every 6 weeks. Patients under ICIs treatment were defined as response group (R) (with partial response or stable disease) or non-response group (NR) (with progression disease) at the time point of clinical evaluation according to the RECIST 1.1 evaluation. The long-term efficacy was evaluated by progression-free survival (PFS) and overall survival (OS). PFS was defined as the time from initiation of ICIs to the radiographic or clinical progression or death from any of the causes according to RECIST (RECIST-PFS) or until to the end of the research. OS was defined as the time from initiation of ICIs to death from any of the causes according to RECIST (RECIST-OS) or the end of the research.

### Statistical analysis

Statistical analyses were performed using R4.2.2, Microsoft Excel (Microsoft Inc., Redmond, Washington). Descriptive analyses were performed with either means ± standard deviation (continuous variables) or interquartile range (IQR) to describe the patient’s characteristics. The association between clinical characteristics and clinical responses were determined by using X^2^ test. The differences between clinical characteristics and vaccination were determined by using Fisher’s test or X^2^ test. Continuous variables were compared by Mann-Whitney test or T-test. Kaplan-Meier curve was drawn, and the Log Rank test was carried out to compare the differences in PFS or OS between the different groups. Cox proportional hazards model was conducted to further identify independent prognostic factors associated with PFS and OS. All results tested were two-tailed and were considered statistically significant if the p-value was less than 0.05.

## Results

### Baseline characteristics of lung cancer patients

Among the total 104 patients who received ICIs or combined with chemotherapy included in the study, 91 were male and 13 were female, with a median age of 67 years. All patients were of 0–2 ECOG PS scores, and 75.96% of patients were of 0–1 ECOG PS scores. There were 68 non-squamous NSCLC patients and 36 squamous NSCLC patients. Fifty-five patients received ICIs for first-line treatment, other patients received ICIs for subsequent-line treatment. 53.85% patients had smoking history. None of patients had a history of COVID-19 infection. Twenty-five (24.04%) patients received COVID-19 vaccination, including 8 patients (7.69%) with three shots, 13 patients (12.5%) with two shots, and 4 (3.85%) with one shot. Moreover, 13 patients received the Sinopharm BBIBP-CorV, 11 patients received Sinovac-CoronaVac vaccine, and only one patient received the CanSino vaccine. Seventy-nine patients (75.96%) did not receive COVID-19 vaccination and were classified as the non-vaccinated group. 103 patients received anti-PD-1 treatment, including Camrelizumab (14, 13.46%), Teripulimab (2, 1.29%), Sintilimab (53, 50.96%), Tislelizumab (5, 4.81%), Pembrolizumab (29, 27.88%), and only one patient received anti-PD-L1 (Durvalumab).

The 104 patients were grouped according to clinical response of ICIs. The response group included the patients of complete remission (CR) (0; 0%), partial remission (PR) (16; 15.39%), disease stabilization (SD) (49; 47.12%). These 65 patients were classified as response group (R), whereas the rest patients (n = 39) with disease progression (PD) were classified as non-response group (NR).

Our results showed that there was no difference between the response and non-response groups in terms of sex, disease history, disease stage, and the treatment line, except ECOG PS score (*p* = 0.015) and COVID-19 vaccination (*p* = 0.005). We found that 22 out of 65 patients (33.85%) received COVID-19 vaccination in the response group, while 3 out of 39 patients (7.69%) vaccinated in the non-response group (Table 1). Moreover, the levels of baseline NLR, fibrinogen and D-Dimer were lower, while albumin were higher in the response group than in the non-response group (Table 2).

**Table 1:** Clinical characteristics of patients with III–IV stage NSCLC

Characteristic	Total (n = 104)	R (n = 65)	NR (n = 39)	*p* value
Age (y, mean ± SD)	67.85 ± 9.76	70.96 ± 10.27	74.49 ± 11.41	0.062
Sex				0.320
Male	91 (87.50%)	59	32	
Female	13 (12.50%)	6	7	
Smoking status				0.839
Smoker	56 (53.85%)	34	22	
Non-smoker	48 (46.15%)	31	17	
ECOG PS score				0.015
0–1	79 (75.96%)	55	24	
> 1	25 (24.04%)	10	15	
Pathology				1
Squamous	36 (34.62%)	23	13	
Non-squamous	68 (65.38%)	42	26	
Disease stage				0.089
III	21 (20.20%)	17	4	
IV	83 (79.80%)	48	35	
Covid-19 vaccination				0.005
Yes	25 (24.04%)	22	3	
No	79 (75.96%)	43	36	
Vaccination doses				**–**
0	79 (75.96%)	43	36	
1	4 (3.85%)	4	0	
2	13 (12.50%)	10	3	
3	8 (7.69%)	8	0	
Vaccine type				–
No	79 (75.96%)	43	36	
CanSino	1 (0.96%)	1	0	
Sinovac–CoronaVac	13 (12.50%)	1	12	
Sinopharm BBIBP-CorV	11 (10.58%)	9	2	
History of COVID-19 infection				–
Yes	0	0	0	
No	104 (100%)	65	39	
Treatment line				0.094
First line	55 (52.89%)	39	16	
Subsequent lines	49 (47.11%)	26	23	
Combined therapy				0.280
Yes	89 (85.58%)	58	31	
No	15 (14.42%)	7	8	
Anti-PD-(L)1 treatment				–
Camrelizumab	14 (13.46%)	9	5	
Pembrolizumab	29 (27.88%)	19	11	
Teripulimab	2 (1.92%)	2	0	
Sintilimab	53 (50.96%)	32	21	
Tislelizumab	5 (4.81%)	3	2	
Durvalumab	1 (0.96%)	1	0	
Treatment efficacy				-
Complete remission (CR)	0 (0%)	0	–	
Partial remission (PR)	16 (15.38%)	16	–	
Stable disease (SD)	49 (47.12%)	49	–	
Progression disease (PD)	39 (37.50%)	–	39	

*R* Response group; *NR* Non-response group

**Table 2:** Analysis of T cell subsets, IgM, Transferrin, NLR, CRP and D-Dimer in stage III–IV NSCLC Patients

Characteristics	R (n = 65)	NR (n = 39)	*p* value
CD3+ (%)	71.09 ± 9.34	67.23 ± 10.76	0.149
CD4+ (%)	41.48 ± 10.38	38.04 ± 11.16	0.226
CD8+ (%)	23.70 (20.02, 28.50)	24.25 (17.23, 33.25)	0.971
CD4/CD8	1.76 (1.26, 2.29)	1.91 (0.91, 2.08)	0.594
CD16 + 56+ (%)	14.80 (9.43, 18.95)	17.50 (11.00, 24.70)	0.122
CD19+ (%)	7.60 (3.93, 12.20)	8.35 (6.00, 11.01)	0.343
IgM (g/L)	0.84 (0.72, 1.11)	0.89 (0.67, 1.12)	0.931
IgG (g/L)	11.85 (9.33, 13.30)	12.60 (11.15, 15.10)	0.167
IgA (g/L)	2.42 (1.92, 2.77)	2.31 (1.98, 3.04)	0.853
IgE (g/L)	75.00 (26.20, 157.00)	41.00 (18.45, 76.80)	0.088
IgM (g/L)	0.84 (0.72, 1.11)	0.89 (0.67, 1.12)	0.931
Fibrinogen	4.06 ± 1.27	4.83 ± 1.40	0.009
Eosinophil	0.14 (0.06, 0.25)	0.11 (0.04, 0.21)	0.180
NLR	3.13 (2.36, 5.93)	4.34 (3.53, 8.94)	0.002
D-Dimer (mg/L)	1.01 (0.56, 1.85)	2.10 (1.13, 3.46)	0.007
Albumin	37.04 ± 5.27	32.21 ± 4.86	0.000
LDH	224.50 (191.15, 300)	330.45 (262, 507.50)	0.001

*R* Response group; *NR* Non-response group; *NLR* Neutrophil-lymphocyte ratio; *IgM* Immunoglobulin M;*IgG* Immunoglobulin G; *IgA* Immunoglobulin A; *IgE* Immunoglobulin E

### COVID-19 vaccination is associated with PFS benefit in lung cancer patient with ICIs treatment

**Fig. 2:**
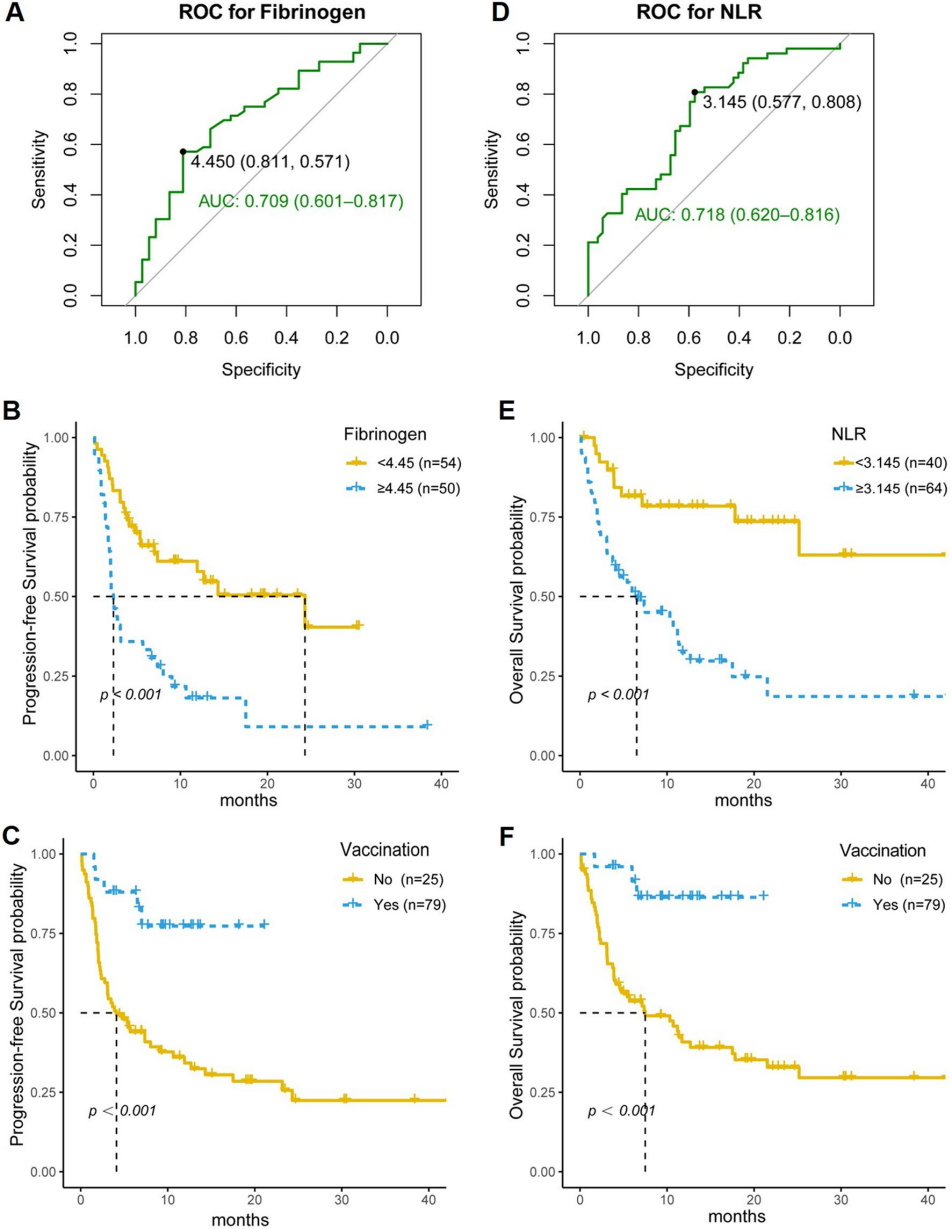
The influence of fibrinogen, NLR or COVID-19 vaccination on PFS or OS. **A** The ROC curves for fibrinogen; **B** The effect of fibrinogen on progression-free survival (PFS); **C** The effect of COVID-19 vaccination on PFS; **D** The ROC curves for NLR. **E** The effect of NLR on overall survival (OS); **F** The effect of COVID-19 vaccination on OS

The median PFS of the 104 patients with stage of III-IV NSCLC was 9.03 months (95% CI 4.76–15.10). Univariate analysis indicated physical status (PS) score (*p* = 0.033), PD-L1 expression (*p* = 0.021), COVID-19 vaccination (*p* = 0.002), albumin (*p* < 0.001), fibrinogen (*p* < 0.001) checked before ICIs treatment were independent prognostic factors for PFS in NSCLC patients with ICIs treatment. Furthermore, the multivariate analysis showed that COVID-19 vaccination (*p* = 0.013), fibrinogen (*p* = 0.008) checked before ICIs treatment were independent prognostic factors for PFS in NSCLC patients with ICIs treatment (Table 3). Notably, the efficacy (AUC = 0.709) of fibrinogen to predict the therapeutic benefit of ICIs with a sensitivity of 81.10% and specificity of 57.1% using fibrinogen < 4.45 g/L as a threshold (Fig. 2A). Kaplan–Meier survival curves demonstrated a close relationship between fibrinogen and the efficacy of ICIs (*p* < 0.001) (Fig. 2B). Moreover, the Kaplan–Meier survival curve showed the vaccinated group (median PFS: not reached) had a significantly better PFS benefit than the non-vaccinated group (median PFS: 4.11 months) (*p* < 0.001) (Fig. 2C).

**Table 3:** Univariate and multivariate Cox regression analysis of clinical characteristics and laboratory indicators on PFS

Characteristics	Univariate analysis			Multivariate analysis		
	HR	CI 95%	*p* value	HR	CI 95%	*p* value
Age	1.005	0.979–1.031	0.700			
Gender	1.256	0.616–2.558	0.530			
Smoking	1.551	0.919–2.620	0.100			
ECOG	1.806	1.047–3.115	0.033	1.441	0.728–2.850	0.294
Treatment line	1.333	0.800–2.220	0.27			
Combined therapy	0.696	0.361–1.344	0.28			
Stage	1.869	0.918–3.804	0.085			
PD-(L)1	0.493	0.271-0.900	0.021	0.572	0.283–1.157	0.120
Vaccination	0.231	0.092–0.579	0.002	0.263	0.091–0.757	0.013
NLR	1.038	1.015–1.061	0.001	1.032	0.992–1.074	0.119
Albumin	0.888	0.849–0.929	0.000	0.955	0.900–1.013	0.127
D-D	1.043	1.000-1.088	0.051			
CD3(%)	0.999	0.958–1.022	0.530			
CD4(%)	0.989	0.959–1.020	0.480			
CD8(%)	1.008	0.977–1.040	0.630			
CD4/CD8	1.003	0.710–1.418	0.990			
CD56(%)	1.021	0.987–1.057	0.230			
CD19(%)	0.980	0.923–1.039	0.500			
IgM	0.892	0.333–2.390	0.820			
IgA	1.207	0.788–1.848	0.390			
IgG	1.040	0.956–1.130	0.360			
IgE	1.000	0.998–1.001	0.390			
Fibrinogen	1.518	1.240–1.857	0.000	1.430	1.096–1.864	0.008

*NLR* Neutrophil-lymphocyte ratio; *IgM* Immunoglobulin M; *IgG*, Immunoglobulin G; *IgA* Immunoglobulin A; *IgE*, Immunoglobulin E; *PFS* Progression-free survival

### COVID-19 vaccination is associated with OS benefit in lung cancer patient with ICIs treatment

The median OS of the 104 patients with stage of III-IV NSCLC was 12.7 months (95% CI 7.49-NA). Univariate analysis indicated physical status score (*p* = 0.004), PD-L1 expression (*p* = 0.026), COVID-19 vaccination (*p* = 0.003), NLR (*p* < 0.001), albumin (*p* < 0.001), and fibrinogen (*p* = 0.001) checked before initial ICIs treatment were independent prognostic factors for OS in NSCLC patients with ICIs treatment. Furthermore, multivariate analysis indicated COVID-19 vaccination (*p* = 0.024), fibrinogen (*p* = 0.004) before ICIs treatment were independent prognostic factors for OS in NSCLC patients with ICI treatment (Table 4). In addition, corresponding parameter cut-off values for maximum AUC areas were calculated from the maximum Youden index, with a cut-off value of 3.145 for NLR (sensitivity = 0.577, specificity = 0.808). Our ROC working curves demonstrated that the AUC areas of NLR predicted the OS of immunotherapy in NSCLC patients as 0.718 (Fig. 2D). There was a difference in survival prognosis (OS) between low and high NLR groups (*p* < 0.001) (Fig. 2E). Moreover, the Kaplan–Meier survival curve showed the vaccinated group (median PFS: not reached) had a significantly better OS benefit than the non-vaccinated group (median PFS: 7.49 months) (*p* < 0.001) (Fig. 2F).

**Table 4:** Univariate and multivariate Cox regression analysis of clinical characteristics and laboratory indicators on OS

Characteristic	Univariate analysis			Multivariate analysis		
	HR	CI 95%	*p* value	HR	CI 95%	*p* value
Age	1.019	0.991–1.048	0.190			
Gender	1.247	0.585–2.658	0.570			
Smoking	1.533	0.877–2.680	0.130			
ECOG	2.322	1.308–4.123	0.004	2.026	0.971–4.227	0.050
Treatment line	1.680	0.967–2.919	0.066			
Combined therapy	0.609	0.312–1.190	0.150			
Stage	1.697	0.798–3.610	0.170			
PD-(L)1	0.483	0.254–0.917	0.026	0.751	0.359–1.573	0.447
Vaccination	0.166	0.052–0.533	0.003	0.186	0.043–0.798	0.023
NLR	1.051	1.028–1.075	0.000	1.060	1.019–1.103	0.004
Albumin	0.880	0.839–0.924	0.000	0.937	0.877–1.001	0.053
D-D	1.017	0.970–1.066	0.480			
CD3	0.986	0.952–1.019	0.380			
CD4	0.988	0.957–1.020	0.470			
CD8	1.005	0.971–1.039	0.790			
CD4/CD8	1.053	0.733–1.512	0.780			
CD56	1.019	0.983–1.056	0.300			
CD19	0.997	0.939–1.059	0.930			
IgM	0.634	0.203–1.978	0.430			
IgA	1.137	0.719-1.800	0.580			
IgG	1.027	0.948–1.114	0.510			
IgE	1.000	0.999–1.001	0.500			
Fibrinogen	1.397	1.138–1.717	0.001	1.301	0.971–1.742	0.070

*NLR* Neutrophil-lymphocyte ratio; *IgM* Immunoglobulin M; *IgG* Immunoglobulin G; *IgA* Immunoglobulin A; *IgE* Immunoglobulin E; *OS* Overall survival

### Subgroup analysis according to ICIs treatment line or patients’ physical condition

Furthermore, COVID-19 vaccinated patients had better physical conditions. We also found that there is a difference of CD4 + T cell (*p* = 0.047) or CD8 + T cell (*p* = 0.049) levels between the non-vaccinated and vaccinated groups. However, there was no significant difference, in terms of CD19 + B cell, CD3 + T cell, CD16 + CD56 + NK cell, IgG, IgA, IgM, IgE, NLR, CRP, and D-Dimer, between the vaccinated and the non-vaccinated group (Table 5).

**Table 5:** Analysis of clinical factors and laboratory factors between the vaccinated and non-vaccinated patients

Characteristic	Non-vaccinated (n = 79)	vaccinated (n = 25)	*p* value
Age (y, mean ± SD)	67.87 ± 10.78	67.76 ± 5.5	0.945
Sex			0.729
Male	68	23	
Female	11	2	
ECOG			0.006
0–1	55	24	
> 1	24	1	
History			1
Squamous	27	9	
Non-squamous	52	16	
Smoking status			0.713
Smoker	42	15	
Nonsmoker	37	10	
Disease stage			0.161
III	13	8	
IV	66	17	
Treatment line			0.004
First line	35	20	
Subsequent lines	44	5	
DCR	54.43%(43)	88%(22)	0.005
ORR	11.39%(9)	28%(7)	0.050
CD3+ (%)	72.06(61.05,77.42)	71.35(62.875, 74.6)	0.850
CD4+ (%)	38.69 ± 11.60	43.71 ± 6.99	0.047
CD8+ (%)	25.23(20.76,30.10)	20.60(16.55,23.75)	0.049
CD4/CD8	1.68(0.97,2.01)	2.11(1.90,3.85)	0.025
CD16 + 56+ (%)	17.31 ± 8.56	13.70 ± 9.21	0.182
CD19+ (%)	7.7(4.8,10.63)	10.97(6.63,15.78)	0.069
IgM (g/L)	0.84(0.71,1.14)	0.85(0.67,1.09)	0.642
IgG (g/L)	12.40(9.80,13.95)	12.30(10.31,13.25)	0.747
IgA (g/L)	2.36(1.82,3.01)	2.51(2.27,2.79)	0.832
IgE (g/L)	38.7(22.13,105.00)	115(23.80,157)	0.449
IgM(g/L)	0.84(0.71,1.14)	0.85(0.67,1.09)	0.642
Transferrin	1.55 ± 0.39	1.79 ± 0.25	0.019
Eosinophil	0.11(0.05,0.205)	0.17(0.06,0.30)	0.057
NLR (g/L)	4.02(2.79,7.42)	3.28(2.63,5.36)	0.177
Fibrinogen (g/L)	4.24(3.29,5.33)	3.90(2.96,4.90)	0.675
D-Dimer (mg/L)	1.38(0.70,3.29)	1.18(0.67,1.63)	0.302

*NLR* Neutrophil-lymphocyte ratio; *IgM* Immunoglobulin M; *IgG* Immunoglobulin G; *IgA* Immunoglobulin A; *IgE* Immunoglobulin E

**Fig. 3:**
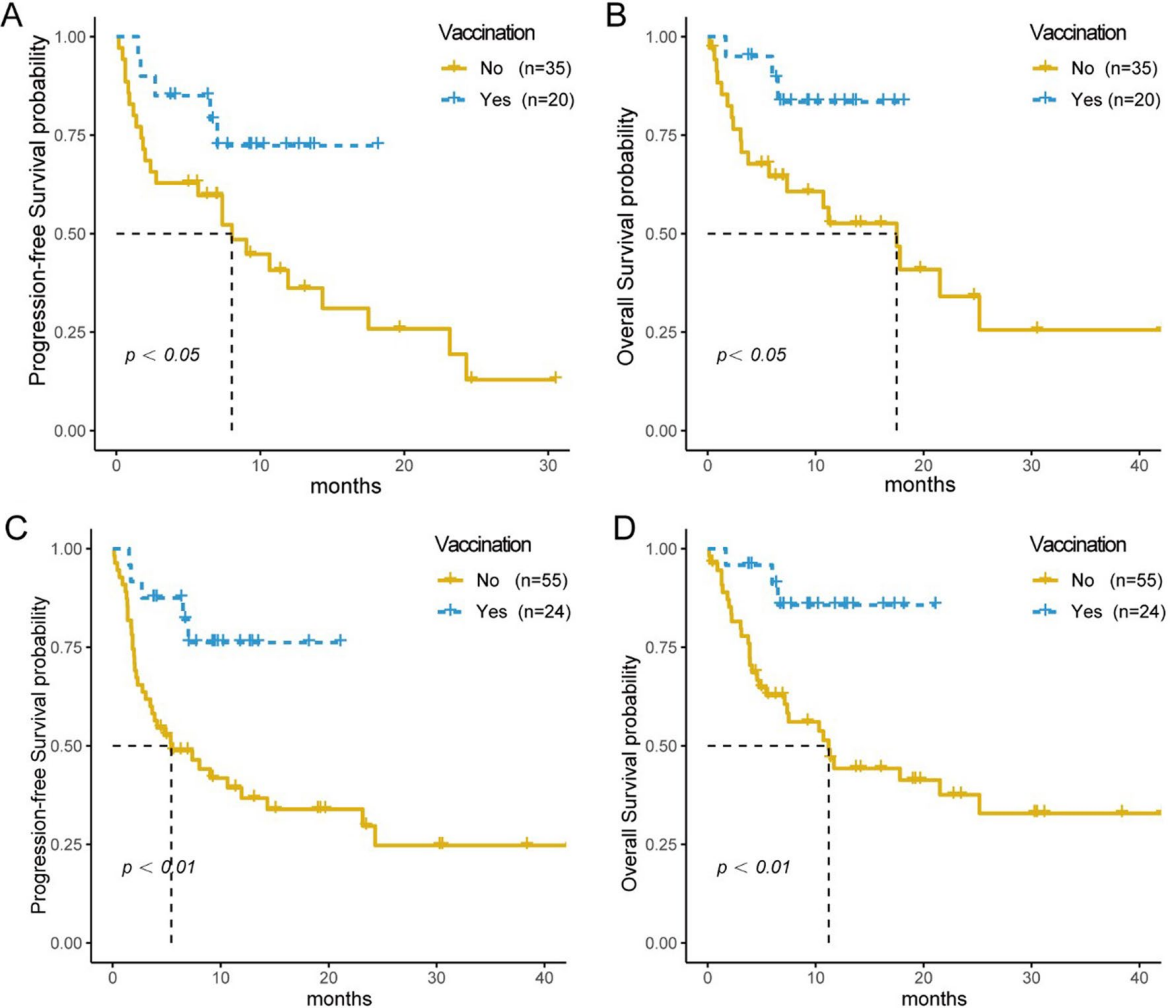
Subgroup analysis according to the ICI treatment lines or physical conditions. PFS (**A**) and OS (**B**) between vaccinated and non-vaccinated groups in patients with first-line ICI treatment; PFS (**C**) and OS (**D**) between vaccinated and non-vaccinated groups in the ECOG 0–1 subgroup

In this study, most of the patients received first-line treatment of ICIs (n = 55, 52.89%), while other patients received subsequent lines treatment of ICIs (n = 49, 47.12%). In order to exclude the influence of treatment lines, 55 patients who received first-line therapy of ICIs were further analyzed. The Kaplan–Meier survival curves showed that the median PFS was not reached in the vaccinated group and 8.02 months in the non-vaccinated group (*p* = 0.036) (Fig. 3A). The median OS was not reached in the vaccinated group and 17.5 months in the non-vaccinated group (*p* = 0.036) (Fig. 3B**).**

Given that the vaccinated patients seemed to have a better physical condition (ECOG 0–1 vs. ECOG 2), we excluded the influence of physical condition, and further analyzed 79 patients of ECOG 0–1. The median PFS was not reached in the vaccinated group, but the median PFS was 5.42 months in the non-vaccinated group (*p* < 0.01) (Fig. 3C); similarly, the median OS was not reached in the vaccinated group, while it was 11.2 months in the non-vaccinated group (*p* < 0.01) (Fig. 3D).

## Discussion

As risk increases for severe COVID-19 infection and associated mortality in patients with immune-compromised cancers [3, 21, 22], they are recommended for vaccination as a priority. Previous studies have shown that COVID-19 vaccination is safe in cancer patients [23, 24], and it may reduce the morbidity and mortality in cancer patients due to COVID-19 infection [25, 26]. There was no increased risk of immune-related adverse events in the patients with influenza vaccine, who received ICIs [27, 28]. Moreover, those who received COVID-19 vaccination are more likely to experience mild immune-related adverse events [20], but mild irAEs following anti-PD-(L)1 treatment may be associated with improved clinical benefit [29, 30]. However, a meta-analysis revealed that cancer patients remained skeptical about vaccination especially for safety concerns, and lack of adherence [31].

Although COVID-19 vaccination was generally suggested to be safe [32], the COVID-19 vaccine-related side-effects were usually mild and did not lead to cessation of cancer treatment, and no difference was found in the risk of irAE between the patients who received or did not receive COVID-19 vaccine [18, 33]. On the other hand, few data are available regarding the influence of COVID-19 vaccination in the context of treatment with ICIs in NSCLC patients. COVID-19 vaccination might increase the immune-related responses due to ICIs therapy [19], but a study suggested that COVID-19 vaccination status does not have a significant influence on patients’ outcomes [34].

Our results showed a better ORR (28.0% vs. 11.39%, *p* = 0.05) and DCR (88.0% vs. 54.43%, *p* = 0.005) in the COVID-19 vaccinated group, compared to the non-vaccinated group. Furthermore, we also found that improved PFS (*p* < 0.001) and OS (*p* < 0.001) in the vaccinated subgroup. Moreover, the vaccinated group demonstrated a benefit survival (PFS or OS) as compared with the non-vaccinated group either in the subgroup with ECOG PS scores 0–1 or in the subgroup with first-line treatment.

Our laboratory findings indicated that CD4 + T cell level was higher in the vaccinated group than that in the non-vaccinated group (*p* = 0.047). Given that tumor immunology influences the response to antitumor therapy including radiotherapy and chemotherapy [35–37], in particular tumor immune-microenvironment(TIME) plays the key role in the response to immunotherapy [38], inadequate T-cell priming, or other T-cell inhibitory immune cells including regulatory T cells (Treg) or myeloid derived suppressive cells (MDSC) can lead to non-response of ICIs treatment [38]. Moreover, in secondary lymphoid organs, CD4 + T cells improve the magnitude and quality of B-cell responses and CTL responses [39]. Thus, COVID-19 vaccine may help promote the activation of CD4 + T cell and reprogramming of the tumor microenvironment.

This study still has a few limitations. It is a retrospective and single-center study. Moreover, due to the limited patient size, further evaluation and stratification with more patients are needed, in particular subgroup analysis. Finally, our study hopefully can raise public awareness of the benefit from COVID-19 vaccination in cancer patients.

## Conclusions

This study demonstrates a potential interaction between COVID-19 vaccination and the efficacy of anti-PD-(L)1 immunotherapy in NSCLC patients, and suggests that NSCLC patients who received anti-PD-(L)1 treatment might benefit from COVID-19 vaccination in terms of survival. Therefore, our data may also suggest a previously unrecognized regulatory potential of COVID-19 vaccination in cancer immunotherapy.

## Data Availability

The datasets used and analyzed during the current study are available from the corresponding author on reasonable request.
